# Granular Hydrogel Composites as Matrices for Engineered Living Materials

**DOI:** 10.1021/acsapm.6c03807

**Published:** 2026-09-25

**Authors:** Samson Oluwagbenga Adelani, Morgan B. Riffe, Jason A. Burdick, R. Konane Bay

**Affiliations:** † Materials Science and Engineering Program, 1877 University of Colorado, Boulder, Colorado 80303, United States; ‡ Department of Chemical and Biological Engineering, University of Colorado, Boulder, Colorado 80303, United States; § BioFrontiers Institute, University of Colorado, Boulder, Colorado 80303, United States

**Keywords:** Bacteria, granular hydrogels, mechanics, engineered living materials, confinement

## Abstract

The design of engineered living materials (ELMs) that integrate active cells offers opportunities to create dynamic, adaptive systems. However, current methods often use bulk hydrogels that can immobilize cells and limit their growth and migration, which affects their overall functionality for use as an ELM. Here, we developed a granular hydrogel composite composed of hydrogel particles (i.e., microgels), *Escherichia coli* (*E. coli*), and a cross-linkable polymer that can be crosslinked with light exposure when desired. We investigated the influence of matrix composition and incubation conditions on microcolony morphology and mechanical properties and observed that the addition of microgels to the composite results in larger bacterial colonies with roughened morphologies and that the granular hydrogel composite compressive modulus is dependent on the matrix composition and incubation conditions. To further demonstrate the potential of this system, we also showed that the granular hydrogel composites can be 3D printed into specified geometries, that the composites can be used for sustained release of bacteria, and that the un-crosslinked matrix supports chemotactic migration, producing cell density gradients that can be fixed in place with crosslinking. Overall, the granular hydrogel composites provide an approach for the fabrication of ELMs.

## Graphical abstract

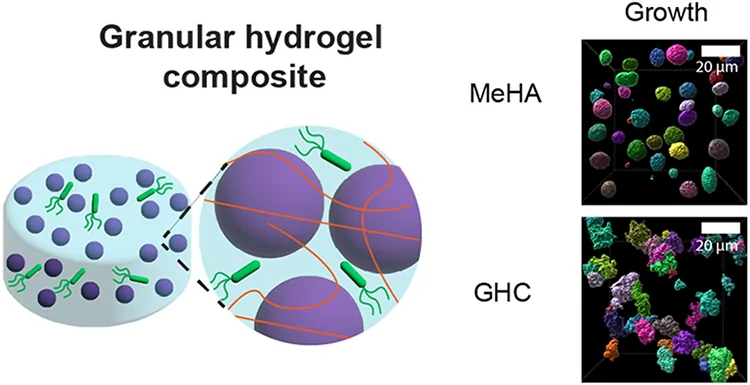

## 1 Introduction

There is a growing demand for responsive and multifunctional soft materials with adaptive, self-healing, and sensing capabilities.^1,2^ These capabilities are vital for emerging applications in soft robotics,^3^ environmental monitoring,^4,5^ and regenerative medicine,^6,7^ where conventional synthetic materials often lack the adaptability and self-repair mechanisms needed for long-term performance.^8,9^ To meet these demands, the emerging field of engineered living materials (ELMs) has recently gained significant interest due to its integration of programmable biological functions into synthetic systems.^10,11^ By incorporating living cells into materials, such as swollen polymer networks (i.e., hydrogels),^12–16^ ELMs harness unique behaviors of microbes to achieve dynamic and programmable material properties. These cell-driven functions have opened new opportunities for applications such as biosensors, self-healing devices, and stimuli-responsive materials for soft robotics.^7,17–20^

The majority of methods for incorporating living cells into ELMs immobilize microorganisms within a “bulk” hydrogel,^17,21,22^ where the hydrogel provides a continuous and uniform aqueous environment that is conducive to efficient nutrient/waste transfer and cell viability.^15,16,23^ Currently, to control the mechanical properties of hydrogel-based ELMs, researchers either increase the bacterial loading during ELM fabrication or tune the crosslinking density of the hydrogel, which can have competing effects. For example, Rivera-Tarazona et al. observed in yeast-loaded ELMs that the addition of yeast in softer matrices (low crosslink density, ∼4 kPa) leads to an increase in compressive modulus and the addition of the cells in stiffer matrices (high crosslink density, ∼40 kPa) leads to a decrease in the compressive modulus. They associate the increase in the modulus in the soft systems to reinforcement of the matrix and the reduction in the modulus to the cells altering the crosslinking reaction.^24^ Beyond impacting the mechanical properties of the ELM, the hydrogel crosslink density can also alter the microcolony volume and protein expression of the cells due to their confinement by the hydrogel matrix.^25^ Thus, researchers are often limited to softer hydrogel matrices to achieve desired functionality, which reduces the potential utility of hydrogel-based ELMs.

Granular hydrogels present one solution to overcome these limitations of bulk hydrogels. Unlike bulk hydrogels, which form a continuous network that restricts microbial movement, granular hydrogels feature an interconnected porous structure with locally rearrangeable particles.^26^ This design enables dynamic interactions between embedded cells and the matrix. For instance, studies have shown that un-crosslinked microgels support bacterial behavior and spatial patterning, such as growth, migration, and self-organization into cell concentration gradients.^27–31^ Despite these advantages, un-crosslinked microgel assemblies lack the cohesion required to form stable, freestanding structures. These microgels can be crosslinked through either interparticle crosslinking or the crosslinking of an interstitial matrix between particles. This has been explored for biomedical applications such as 3D printing and tissue engineering.^28–30^

Here, we fabricated granular hydrogel composites composed of *Escherichia coli* (*E. coli*), photo-crosslinkable hyaluronic acid (HA), and crosslinked acrylic acid/alkyl acrylate copolymer microgels. Before crosslinking, the jammed microgel network provides an interconnected interstitial space that allows for microbial growth and motility, and subsequent crosslinking of the HA matrix stabilizes the composites and locks in the desired structure. To explore the potential parameter space within this culture system, we varied the timing of crosslinking and incubation time and monitored the microcolony morphology and size and the resultant compressive modulus. We also explored the use of this system to control the structure of the granular hydrogel composites through 3D printing and spatial patterning of cells. Overall, our results show that granular hydrogel composites can serve as potential scaffolds for ELMs, which could advance the development of next generation of ELMs.

## 2 Materials and Methods

### 2.1 Materials

Crosslinked acrylic acid/alkyl acrylate copolymer microgels (Carbomer 980NF) were obtained from Lubrizol (Wickliffe, OH, USA). Methacrylic anhydride, lithium phenyl (2,4,6-trimethylbenzoyl) phosphinate (LAP), 10 M sodium hydroxide (NaOH), and Lennox Luria Broth (LB) were purchased from Sigma-Aldrich (St. Louis, MO, USA). Sodium hyaluronate (HA, 70 kDa) was purchased from LifeCore Biomedical (Chaska, MN, USA).

### 2.2 Bacteria Strain and Culture Conditions

*E. coli* W3110 that constitutively expresses green fluorescent protein (GFP) were harvested from a frozen bacterial glycerol stock (stored at −70 °C) by obtaining an ice crystal with an inoculating loop and swirling it into 3 mL of sterile LB. This was incubated overnight at 37 °C and shaking at 220 rpm. The next day, 200 μL of the overnight culture was transferred into 3 mL of fresh LB and incubated at 37 °C, shaking at 220 rpm for ∼3 h, until the optical density (OD) at 600 nm reached 0.5.

### 2.3 Synthesis of Methacrylated Hyaluronic Acid (MeHA)

Sodium hyaluronate (*M*_W_ = 70 kDa) was methacrylated as previously reported.^32^ Briefly, HA was dissolved in deionized water and cooled on ice, and methacrylic anhydride was added dropwise while maintaining the pH at 8.5–9.5 using 1 N NaOH. The reaction was allowed to proceed for 4 h under continuous pH adjustment, followed by stirring overnight at room temperature. The product was then precipitated in cold acetone, redissolved in deionized water, and dialyzed against water for 24 h. After dialysis, MeHA was frozen at −70 °C and lyophilized. The degree of methacrylation was determined to be 70% degree of substitution by dissolving MeHA at 1 wt % in D_2_O and analyzing via ^1^H NMR spectroscopy (400 MHz, Bruker, Figure S1).

### 2.4 Preparation of Granular Hydrogel

The granular hydrogel was prepared in a biosafety cabinet by suspending crosslinked acrylic acid/alkyl acrylate copolymer microgels at defined concentrations (0.6–0.8 wt %) in 150 mL of 2% LB. The mixture was homogenized for 2 min using a stand mixer, and the pH was adjusted to 7.4 by adding 10 M NaOH. As the pH increased, the particles swelled (5–10 μm diameter),^33^ forming a jammed matrix with an internal mesh size of ∼40–100 nm.^34^ The jammed matrix was transferred to a 50 mL centrifuge tube using a sterile syringe and centrifuged at 1449*g* for 3 min to remove air bubbles. It was then left at room temperature for 48 h to confirm that no contamination occurred, which would be visible as suspended microcolonies. A second centrifugation under the same conditions was then performed before use. All LB medium was sterilized by autoclaving before use. Sample preparation, cell encapsulation, and culture handling were performed in a biosafety cabinet using sterile techniques to minimize contamination.

### 2.5 Fabrication of Microbial Granular Hydrogel Composite

Granular hydrogel composites were prepared in a biosafety cabinet, using a 30 mL sterile plastic syringe by mixing equal volumes of the swollen granular hydrogel and a MeHA precursor solution (1:1 ratio), both formulated in 2% LB. The concentration of the granular hydrogel was varied between 0.6 and 0.8 wt % to modulate particle packing. The MeHA precursor solution was prepared by dissolving MeHA at 3 wt % in 2% LB, supplemented with 0.1 wt % lithium phenyl-2,4,6-trimethylbenzoylphosphinate (LAP) as the photoinitiator. Before mixing, the granular hydrogel matrix was centrifuged at 1449*g* for 2 min to remove bubbles that had formed during storage. The MeHA–LAP solution was then added to the granular hydrogel and gently mixed by pipetting to form a uniform composite, ensuring even distribution of the interstitial matrix.

The interstitial pore size before crosslinking was measured by incorporating 100 nm carboxylated fluorescent polystyrene nanoparticles into the mixture and imaging the composites using confocal microscopy. Particle motion was tracked using MATLAB code, following established methods.^33^ Briefly, the MATLAB script tracked the nanoparticles’ trajectories using the classic Crocker-Grier particle tracking algorithm, which identifies each particle and tracks its center of mass across time-lapse frames. From the particle trajectories, the code computed the mean squared displacement (MSD) for each nanoparticle. For constrained diffusion in porous media, the MSD is expected to exhibit an initial regime of free diffusion at short time and length scales, followed by a transition to subdiffusive scaling at longer scales due to confinement imposed by the surrounding particle network. We estimate the characteristic pore size by identifying the length scale at which the MSD deviates from free-diffusive behavior and enters the subdiffusive regime. The pore size for each tracked nanoparticle was calculated by adding this transition length scale to the nanoparticle diameter. Repeating this analysis for all tracked particles yielded a pore-size distribution. The distribution was converted to a cumulative distribution function (CDF). An exponential function B*exp­(-*a*/*A*) was fit to the (1- CDF) data in the range of 0 < CDF < 1. We report the characteristic pore size as (*A* + *a*_0_), where *a*_0_ is the largest pore size with CDF = 0, and A is the exponential decay. Representative particle trajectories and ensemble-averaged MSD curves are provided in Figure S2 of the Supporting Information.

For microbial granular hydrogel composites, *E. coli* cultures were grown to midexponential phase (OD_600_ ≈ 0.6) and added to the MeHA-LAP solution to achieve a final cell concentration of 3.18 × 10^6^ cells/mL in the composite. The cell-laden GHCs were divided into two growth conditions based on the timing of photo-crosslinking: GHC grown crosslinked and GHC grown un-crosslinked. GHC grown crosslinked samples were photo-crosslinked immediately after fabrication and subsequently incubated at 37 °C under stationary conditions for 24 or 48 h. In contrast, GHC grown un-crosslinked samples were incubated in the un-crosslinked state at 37 °C under stationary conditions for 24 or 48 h, allowing bacterial growth and redistribution within the granular matrix and then photo-crosslinking prior to all characterization. A beaker of water was maintained in the incubator throughout the incubation period to provide a humid environment and minimize water loss from the samples. Photo-crosslinking was performed by exposing the composites to UV light (λ = 365 nm, 5 mW/cm^2^, Omnicure S2000 Elite) for 5 min.

### 2.6 Rheological Characterization of Granular Hydrogel Composites

The rheological behavior of the granular hydrogel composite precursors was characterized using an oscillatory shear rheometer (AR2000, TA Instruments) fitted with a 20 mm parallel-plate geometry and a fixed gap of 100 μm. A strain sweep was performed from 0.1% to 1000% strain to determine the linear viscoelastic region. Flow properties, including viscosity, yield stress, and shear stress, were evaluated using a steady-state shear rate sweep from 10^–4^ to 10^2^ s^–1^. Photorheology experiments were conducted using a Discovery HR20 rheometer (TA Instruments) with an 8 mm parallel plate geometry. To prevent dehydration during measurement, mineral oil was applied around the edges of the sample. Measurements were carried out at room temperature with a fixed strain of 0.1% and a frequency of 1 Hz. A UV light source (OmniCure S2000 Elite, Excelitas Technologies, Waltham, MA, λ = 365 nm, 5 mW/cm^2^) was used for photoinitiation. Each run consisted of a 1 min dark baseline, followed by 5 min of UV exposure. The plate gap was set to 1 mm for all photorheology tests.

### 2.7 Compressive Modulus

Granular hydrogel composites were cast and photo-crosslinked in cylindrical molds (8 mm diameter × 4 mm height) before testing. Unconfined compression tests were performed at room temperature using a Texture Analyzer XT Plus (Texture Technologies Corp.) fitted with a 5 kg load cell. Samples were compressed at a constant rate of 0.05 mm/s while force–displacement data were recorded. The compressive moduli were extracted from the stress–strain curves between 10% and 20% strain. At least three independent samples were tested for each condition.

### 2.8 3D Printing of Structures

A 3D model was designed in Autodesk Fusion 360 and 3D printed using a Replistruder. Briefly, the Replistruder was assembled by removing the stock filament extruder and heating block from a commercial 3D printer and replacing them with a syringe-based extrusion module. Following published protocols from Tashman et al.^35^ The motor mount, plunger holder, and carriages were fabricated from 3D-printed components, and the stepper motor was used to drive the syringe plunger. This configuration effectively converted the printer into a programmable syringe pump capable of depositing hydrogel inks with a precise, computer-controlled flow rate. Further modifications were made to the Replistruder to enable a Luer-lock syringe. Granular hydrogel composite precursors were loaded into dark 3 mL syringes fitted with a 20G needle to prevent premature photo-crosslinking. The syringe and needle were mounted in the printer, and deposition was performed under UV light irradiation with a wavelength of 365 nm and an intensity of 5 mW/cm^2^ to induce crosslinking during printing at room temperature. Print fidelity was evaluated using five 25 mm printed lines imaged immediately after deposition. Width retention was calculated from the mean filament width relative to the needle inner diameter (0.660 mm), and length retention from the measured length relative to the programmed length (25 mm), with 100% representing exact dimensional retention.

### 2.9 Quantification of Cell Concentration in the Granular Hydrogel Composite

The cell concentration in the granular hydrogel composite was quantified via colony-forming units (CFU) assays following enzymatic degradation and mechanical disruption. The samples were first gently washed in sterile PBS to remove residual media and loosely attached cells, then transferred into sterile tubes containing 1.5 mg/mL hyaluronidase solution. Samples were incubated at 37 °C for 60 min with intermittent vortexing to promote enzymatic degradation of MeHA. The resulting suspension was further mechanically disrupted by vortexing and pipetting to ensure complete release of the cells from the crosslinked granular network. An aliquot of the solution was diluted into various dilutions and plated on LB-agar plates. Plates were incubated at 30 °C overnight, and the colonies from the plate were counted.

### 2.10 Quantification of Cell Release

Granular hydrogel composites laden with 3.18 × 10^6^ cells/mL of *E. coli* were cast and photo-crosslinked in cylindrical molds (8 mm diameter × 4 mm height). The samples were incubated in 10 mL of LB media using a 50 mL centrifuge tube at 37 °C with constant shaking at 220 rpm. Aliquots from the surrounding medium were collected at 2, 24 and 48, and 72 h, serially diluted 1000-fold, and plated on an LB agar plate. Plates were incubated overnight at 37 °C for 24 h, and colony-forming units from the plates were counted. The samples were removed from the medium every 24 h, washed three times with Phosphate-buffered saline (PBS) solution, and transferred back into fresh medium before further incubation.

### 2.11 Quantification of Colony Morphology

Granular hydrogel composites containing an initial *E. coli* concentration of 3.18 × 10^6^ cells/mL were prepared using two protocols. In the first, hydrogels were photo-crosslinked immediately after cell encapsulation (0 h). In the second, cell-laden composites were incubated at 37 °C for 24 or 48 h before photo-crosslinking to allow bacterial proliferation before crosslinking. Homogeneous 3 wt % MeHA hydrogels containing the same initial bacterial concentration and photo-crosslinked immediately after encapsulation served as controls. Samples were imaged every 24 h for up to 48 h using a Nikon A1R laser scanning confocal microscope with a 20× objective (512 × 512 pixels, z-stacks up to ∼220 μm, 0.25 μm step size). Confocal image stacks (ND2 files) were analyzed for both individual colony volume and total bacterial volume fraction. Individual bacterial colonies were segmented and reconstructed as three-dimensional surfaces using the Surfaces module in Imaris (Oxford Instruments, Version 11.1, Switzerland). The volume (μm^3^) of each segmented colony was automatically calculated and used for colony volume analysis. For bacterial volume fraction analysis, the bacterial fluorescence was segmented following background subtraction and Gaussian smoothing. The calibrated voxel dimensions (pixel size in the *x*–*y* plane and *z*-spacing) were obtained from the image metadata. The total bacterial volume (*V*_colony_) was calculated from the number of segmented bacterial voxels multiplied by the calibrated voxel volume. The bacterial volume fraction was then calculated as

1 $$\phi_{\mathrm{cell}}=\frac{V_{\mathrm{colony}}}{V_{\mathrm{stack}}}$$

where *V*_stacks_ is the total confocal imaging volume determined from the calibrated image dimensions.

Colony morphology was quantified from the segmented bacterial colonies. For each colony, the boundary coordinates were extracted and the centroid was determined. The radial distance from the centroid to each boundary point was calculated, and the mean colony radius was determined. Boundary roughness was quantified using the arithmetic average roughness (*R*_a_), calculated as the average absolute deviation of the radial distance from the mean radius (*R*_a_):

2 $$R_{a}=\frac{1}{N}\sum_{i=1}^{N}\left|r_{i}-\mathrm{r̅}\right|$$

And the root-mean-square roughness (*R*_q_) was calculated as

3 $$R_{q}=\sqrt{\frac{1}{N}\sum_{i=1}^{N}{\left(r_{i}-\mathrm{r̅}\right)}^{2}}$$

where *r*_*i*_ is the radial distance from the colony centroid to the *i* ^th^ boundary point, r̅ is the mean colony radius, and *N* is the total number of boundary points. Colony volume and roughness were quantified for all segmented colonies within each image stack.

### 2.12 Chemotactic Patterning

A granular hydrogel composite precursor containing 0.7 wt % microgel was introduced into a sterilized 10 mm × 45 mm cuvette. The cuvette was filled to about half its height. A single 12 mm line of concentrated cells (9 × 10^10^ cells/mL) was printed into the center of the granular hydrogel using a 3D Lulzbot Mini 2 printer, which was modified by replacing the print head with a Replistruder designed by following protocols from Tashman et al.^35^ The cuvette was later moved into the imaging chamber, and time-lapse images were taken every 30 min for 24 h using the Nikon D350 camera coupled with a Thorlabs long 6.5X zoom lens with 12 mm fine focus. The final structure of the composite was crosslinked using UV light source (OmniCure S2000 Elite, Excelita Technologies, Waltham, MA, λ = 365 nm, 5 mW/cm^2^ for 5 min) and then released from the cuvette mold.

### 2.13 Statistical Analysis

All quantitative data are reported as mean ± standard deviation, and the numbers of samples used are stated within figure captions. Statistical analyses were performed using a one-way analysis of variance (ANOVA) to assess the differences among groups, followed by Tukey’s post hoc test for multiple comparisons. Statistical significance was defined as *p* < 0.05, with significance level annotated as follows: *p < 0.05, **p < 0.01, ***p < 0.001.

## 3 Results and Discussion

### 3.1 Design of the Granular Hydrogel Composite

Granular hydrogel composites (GHCs) were composed of commercially available crosslinked acrylic acid/alkyl acrylate copolymer microgels, a photo-crosslinkable MeHA interstitial matrix, and *E. coli*, as illustrated in Figure 1. Both synthetic components are considered cytocompatible and MeHA has been widely used in tissue engineering applications.^36–39^ The microgels are jammed and exhibit a pore space that allows for cells to grow and move within the interstitial space.^40,41^ The photo-crosslinkable MeHA serves a distinct functional since it infiltrates the interstitial space between the microgels and can be used to fix the cells in place when crosslinked with light. For the majority of our granular hydrogel composites, the microgel and MeHA concentrations were 0.7 and 3 wt %, respectively, resulting in an average interstitial pore size of ∼0.58 ± 0.01 μm before crosslinking, which is slightly larger than the average cell width of *E. coli* (0.5 μm)­(Figure S2).^42^ This size is expected to allow cell migration throughout the matrix.^34^ Using this matrix platform, we investigated how microcolony size and morphology, the composite mechanics, and the spatial organization of *E. coli* depend on the matrix environment (e.g., presence of interstitial crosslinking) and incubation time.

**Figure 1:**
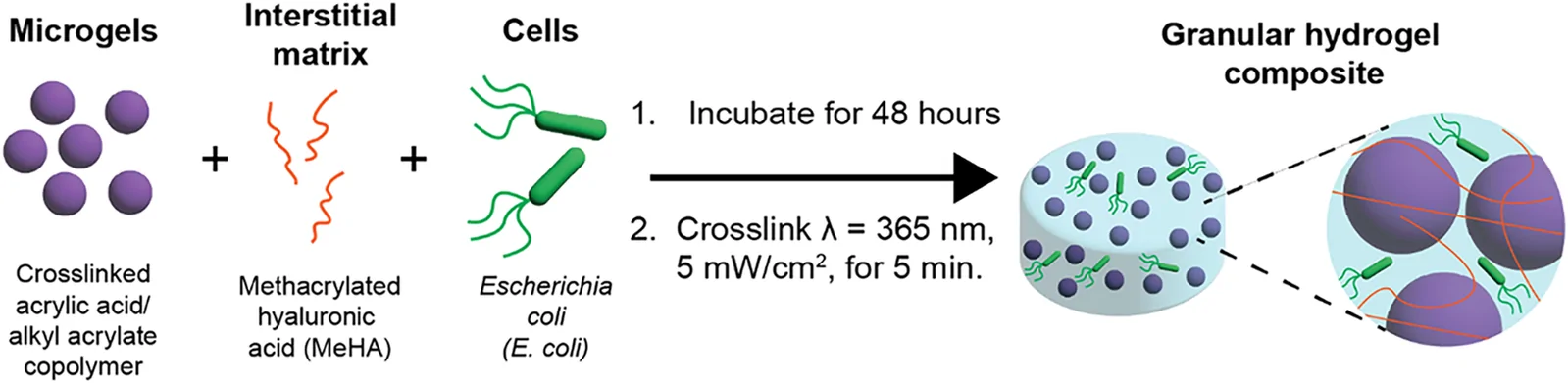
Schematic of the fabrication process of granular hydrogel composites. Granular hydrogel composites are formed through the mixing of microgels (crosslinked acrylic acid and alkyl acrylate copolymer), an interstitial matrix (methacrylated hyaluronic acid, MeHA), and *E. coli*. The mixture is then incubated for up to 48 h to allow for cell growth and can be crosslinked.

### 3.2 Colony Size and Morphology Depend on Matrix

The growth of microbial colonies within hydrogels depends on the mechanical and spatial constraints of the embedding matrix. To investigate the impact of matrix composition, we compared two hydrogel formulations: a 3 wt % MeHA hydrogel and a GHC composed of 0.7 wt % microgels and 3 wt % MeHA. In both cases, the hydrogels were photo-crosslinked immediately after cell encapsulation to isolate the effect of matrix composition on bacterial growth. Representative colony morphologies and sizes as a function of incubation time are shown in Figures 2 and S3.

**Figure 2:**
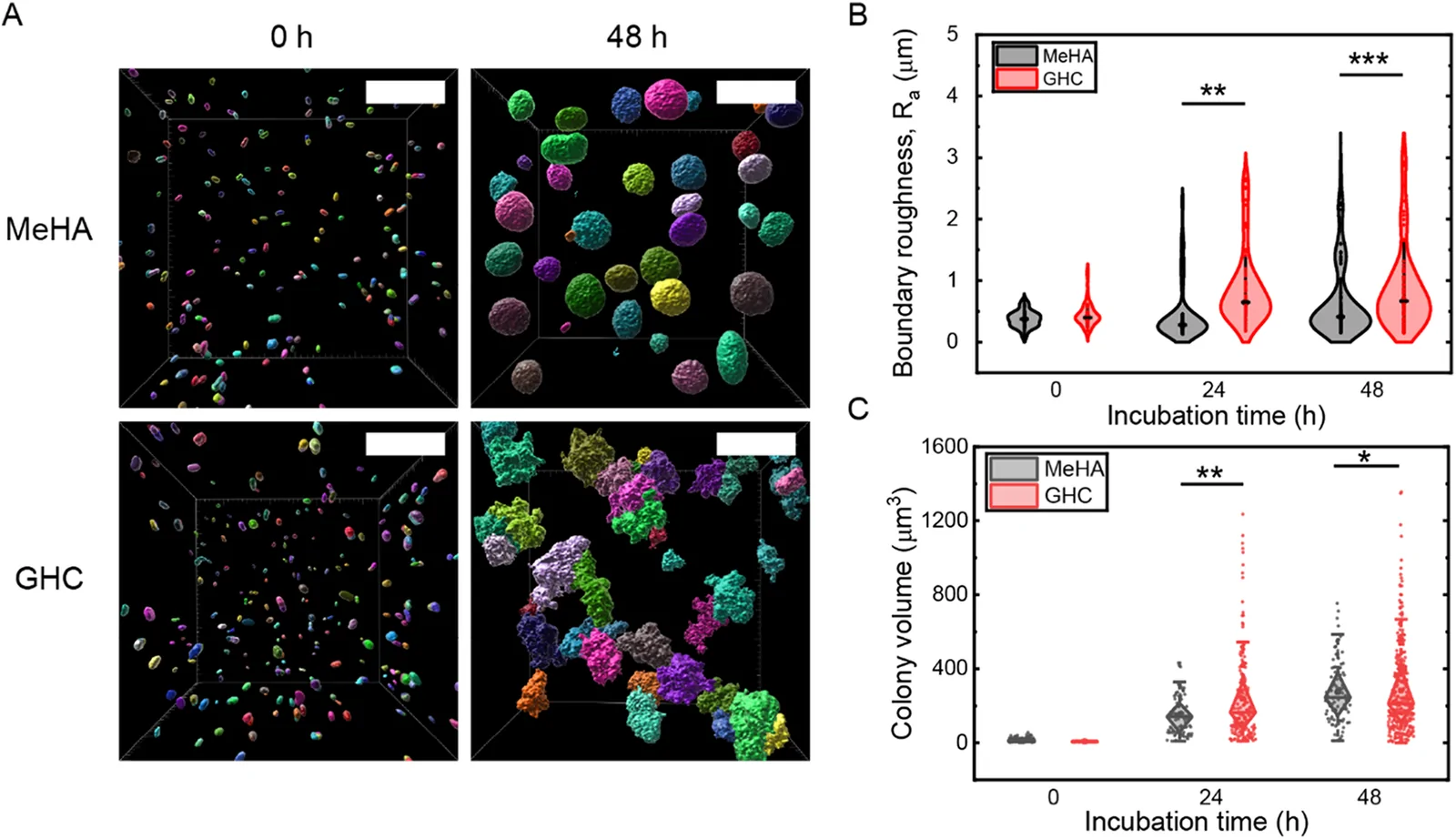
Colony size and morphology depend on matrix structure. (A) Representative three-dimensional reconstructions of the corresponding bacterial colonies generated from confocal z-stacks using Imaris. Individual colonies are displayed as segmented surfaces to visualize colony morphology and growth. Scale bars = 20 μm. (B) Quantification of colony boundary roughness (*R*_a_). Colonies cultured in GHCs exhibited significantly greater boundary roughness than those cultured in MeHA hydrogels at both time points, indicating the development of more irregular colony morphologies within the granular matric (C) Quantification of colony volume obtained from three-dimensional image analysis. Colonies cultured in GHCs exhibited significantly larger volumes than those cultured in homogeneous MeHA hydrogels 0 to 48 h. Data are presented as violin plots with overlaid diamond plots indicating the median and the 10th and 90th percentiles; whiskers indicate the fifth and 95th percentiles. A total of 88–405 colonies from three biological replicates were analyzed. Statistical significance was determined by one-way ANOVA followed by Tukey’s multiple comparisons test (*p* < 0.05, *p* < 0.01, *p* < 0.001).

At 0 h of growth, only single cells were visible in both MeHA hydrogels and GHCs (Figure 2A). After 24 and 48 h of incubation, clear differences in colony morphology emerged between the two matrices (Figures 2A and S3). *E. coli* colonies grown in crosslinked MeHA without microgels have radially smooth morphologies (arithmetic mean roughness, *R*_a_ (24 h) = 0.419 ± 0.09 μm, *R*_a_ (48 h) = 0.651 ± 0.03 μm) consistent with previously reported microcolonies.^43–45^ While *E. coli* colonies grown in crosslinked GHC developed irregular, roughened morphologies (*R*_a_ (24 h) = 0.851 ± 0.02 μm, *R*_a_(48 h) = 0.87 ± 0.18 μm). We observed the same trends in root-mean-square roughness (*R*_q_) as with *R*_a_ (Figure S4). The differences in colony morphology likely arise from the distinct physical environments provided by the two matrices. In MeHA, the continuous crosslinked network provides elastic resistance to colony growth;^43,46,47^ whereas, in the GHC, the addition of microgels alters the MeHA network, potentially reducing the elastic resistance of the network to colony growth leading to the roughened morphologies. This is supported by the observations of reduced compressive modulus with the addition of the microgels (Figure S5). Similar roughened morphologies have been observed in un-crosslinked granular hydrogels where the interstitial pore size was less than the cell width.^48^ Those roughened morphologies were associated with nutrient depletion at the core of the colony, inducing a morphological instability and colony boundary roughening.

Beyond colony roughness, we also observed an increase in colony volume with incubation time for both MeHA hydrogels and GHCs. Colony volumes were significantly lower in MeHA hydrogels than in GHCs (*V*_colony_(24 h) = 139 ± 46 μm^3^, *V*_colony_(48 h) = 274 ± 63 μm^3^) compared to GHCs (*V*_colony_(24 h) = 270 ± 11 μm^3^, *V*_colony_(48 h) = 297 ± 31 μm ^3^) at both 24 h (*p* < 0.01) and 48 h (*p* < 0.05) (Figure 2C). We attribute the difference between MeHA and GHCs to differences in elastic resistance between the two matrix compositions. We note due to difficulties in labeling the microgels, we cannot currently resolve the spatial relationship between bacterial colonies and individual microgels, and do not know how the colonies are interacting with the microgels. Therefore, while the reduced mechanical confinement of the granular hydrogel facilitates the formation of larger colonies, nutrient depletion within these larger colonies may also contribute to the boundary roughness observed. Collectively, these findings suggest that bacterial colony morphology in GHCs is governed by the combined effects of matrix structure, local mechanical confinement, and nutrient availability during colony growth.

### 3.3 Mechanics of the Granular Composites

The compressive moduli of the GHCs depend on the growth environment and incubation time. The incorporation of bacteria into MeHA hydrogels resulted in a reduction in compressive modulus, similar to the observed reduction in the compressive modulus of GHCs when microgels were added to MeHA (Figures 3A and S5). These observations are consistent with prior findings where hydrogel networks with dispersed particles or cells have disrupted crosslinking.^49–51^ Here, we examined how the compressive modulus of crosslinked MeHA hydrogels and GHCs changed when the embedded cells were grown in crosslinked and un-crosslinked state (Figure 3A). For cell-laden grown un-crosslinked, all compression testing was done after the GHC were crosslinked.

**Figure 3:**
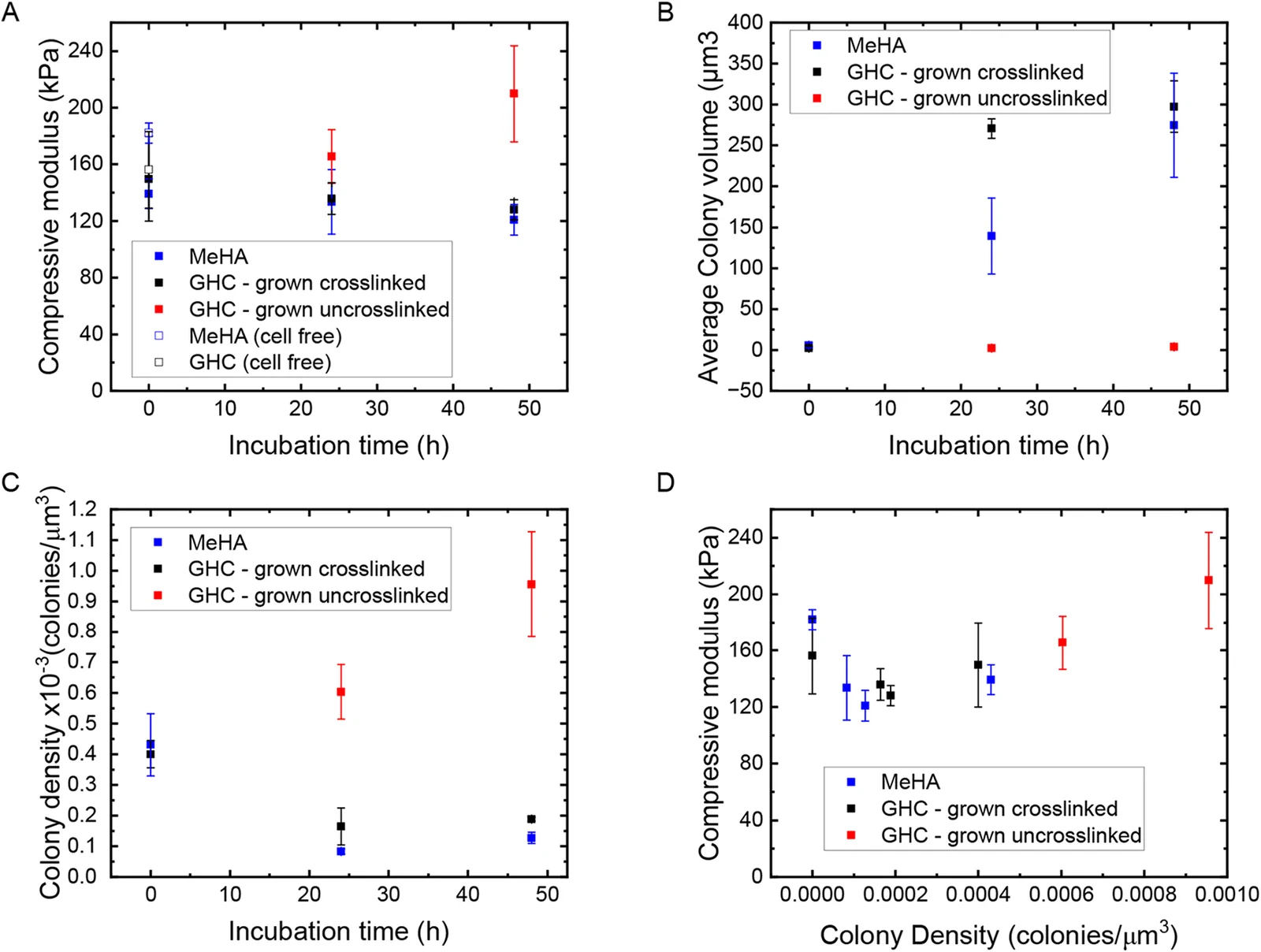
Effect of growth conditions on compressive modulus of granular hydrogel composites. (A) Compressive modulus of granular hydrogel composite MeHA, GHC grown crosslinked, and GHC grown un-crosslinked prior to photo-crosslinking as a function of incubation time. GHC and MeHA were photo-crosslinked before incubation, whereas GHC grown un-crosslinked was incubated in the un-crosslinked state and photo-crosslinked at the indicated time point prior to mechanical testing. (B) Colony volume fraction as a function of incubation time. (C) Colony density, defined as the number of colonies per unit sample volume, as a function of incubation time. (D) Relationship between compressive modulus and colony density. Data are presented as mean ± SD. Compressive modulus measurements (A, D) were obtained from *n* = 3–5 independent hydrogel samples per condition. Bacterial volume fraction and colony density measurements (B, C) were obtained by confocal microscopy from *n* = 3 independent hydrogel samples per condition.

For MeHA hydrogels and GHCs grown crosslinked, the compressive modulus did not significantly change with incubation time, which is surprising since the colonies within the matrices have very different morphologies (Figure 2A). While for cell-laden GHC grown un-crosslinked, we observed an increase in compressive modulus with increasing incubation time. Previous work has shown that when cells are embedded in high stiffness matrices, the modulus decreases.^24,51^ This is opposite from what we measured, where the modulus of MeHA matrix is 187 kPa. Our observation suggests that the time of crosslinking during incubation can have a large influence on the compressive modulus. Given the observed differences in compressive modulus with incubation time and growth environment, we performed confocal microscopy across the conditions investigated. We observed that three growth environments (MeHA, GHC grown crosslinked, GHC grown un-crosslinked) supported bacterial growth in two different ways (Figure S3B). In the crosslinked conditions, the average colony volume increased, but the number of colonies per sample volume (colony density) decreased. While in the un-crosslinked conditions, the colony volume did not change, the bacteria remained as small colonies or individual cells, but the number of colonies increased with incubation time (Figures 3B and S9). To understand how these differences in bacterial growth behavior alters the compressive modulus, we approached our compressive modulus observations through a composite lens. Specifically, we treated the material as a three-phase composite consisting of a MeHA matrix, microgels, and bacterial colonies. In composites, the mechanical response depends not only on the intrinsic properties and relative amount of the components, but also on the size, distribution, and interaction of each component with the surrounding matrix.^52^ In our system, the modulus of *E. coli* is >1 MPa,^53^ elastic modulus of the microgel is <1 kPa,^54^ elastic modulus of MeHA is 182 ± 7 kPa, and the composition of MeHA and microgels were held constant across all the conditions.

Thus, the only differences in each conditions studied was the colony volume and the colony distribution. We note that for the comparison below, we used an approximate average colony volume and approximate colony distribution as the samples used for compressive testing and confocal microscopy were different samples to prevent drying effects that could impact either measurement. For the colony volume, increasing the colony volume within the matrix increased both the colony size and volume fraction. From previous polymer composite literature, increasing the size of a microparticle (bacterial colony) with matrix at a constant volume fraction either decreases or does not impact the compressive modulus.^55,56^ While increasing the volume fraction of microparticles, an increase or decrease in the compressive modulus depends on the stiffness of the surrounding matrix.^24,51^ Specifically, previous work has shown that increasing the volume fraction of cells within a soft matrix (4 kPa) increased the compressive modulus,^24^ while the opposite effect has been observed for cells embedded in stiff matrices (115 kPa).^51^ We did not observe any trends when comparing the normalized modulus to the approximate average colony volume or volume fraction when comparing all three growth conditions (Figure S7A). Specifically, for GHCs and MeHA hydrogels grown crosslinked, we observed that the compressive modulus remained constant as the approximate colony volume and volume fraction increased. These results agree with the previous literature on microparticle size having no effect on the modulus.^56^ For GHCs grown un-crosslinked, we observed an increase in the compressive modulus as the approximate volume fraction increased and average colony volume remained constant with incubation time (Figure S8).

The main difference between the crosslinked and un-crosslinked conditions is the colony volume. For the crosslinked case, there are multiple cells within one colony, while in the un-crosslinked condition, the majority of the colonies are single cells. The smaller colonies are because the cells can grow and redistribute within the available interstitial space before the network is crosslinked. Thus, the spatial distribution of the cells is different between the two crosslinking conditions. In the crosslinked conditions, the colonies can be considered clustered microparticles and in the un-crosslinked condition, the colonies can be considered dispersed microparticles. The spatial organization of micro/nanoparticle within a polymer matrix can have a large influence on the material properties.^57,58^ Specifically, nanocomposites with well-dispersed particles exhibited a greater increase in modulus than those containing clustered particles. Thus, in our system, we quantify this dispersity in spatial organization as the colony density (number of colonies within the sample volume), where higher colony density indicates a disperse organization of the cells. As such, we plotted the compressive modulus versus the approximated colony density for all growth conditions. For the cell-laden materials, we observed an increase in compressive modulus with increasing approximated colony density (Figures 3D and S7B). We do note that the smallest size scale of the cells is ∼500 nm, which is closer in size to a microparticle,^42^ but our results suggest that these individual cells could impact the properties similar to synthetic nanoparticles within a polymer matrix. Our results indicate that the growth environment determines how bacteria colonies are spatially organized when the network is fixed, and this organization is associated with the resulting mechanical response of the composite.

### 3.4 3D Printing and Cell Release

ELMs often require specified macroscopic geometries for applications such as soft robotics and biosensing. Thus, we demonstrate that we can 3D print our granular hydrogel materials. To evaluate the printability of the GHC, cell-laden formulations were fabricated by extrusion printing with simultaneous photo-crosslinking, allowing the deposited structures to retain their designed geometries immediately after printing (Figure 4A). The un-crosslinked GHC exhibited shear-thinning behavior and a well-defined yield stress thus making extrusion 3D printing a viable option (Figure S10). The 3D printing was performed using a modified extrusion printer.^35^ We quantified the print fidelity by comparing the width and length retention of the printed lines. Immediately after printing, the lines exhibited a width retention of 122.5 ± 9.5% and a length retention of 107.3 ± 2.1% (mean ± SD, *n* = 5), demonstrating that the GHC can be fabricated into printed structures with high geometric fidelity (Figure S11). We associated the slight increase above 100% to spreading of the extruded filaments immediately following deposition onto the substrate. Beyond print fidelity, the combination of a jammed microgel structure and a crosslinked matrix supported microbial growth as observed where the opacity of the cell laden 3D printed construct decreased compared to the cell free construct (Figure 4). We also observed a slight change in the morphology of the crosslinked structures during incubation. Specifically, after 24 h of growth in a bath of liquid growth medium, the cell-laden construct exhibited visible deformation, swelling, and localized bulging/roughening, whereas the cell-free controls visibly remained slightly smoother. No further visible changes to the geometry after 24 h of growth were observed (Figure 4). We attribute these initial changes to internal stresses generated by the expanding bacterial colonies within the matrix, with growth-induced pressures driving outward expansion (Figure 4).

**Figure 4:**
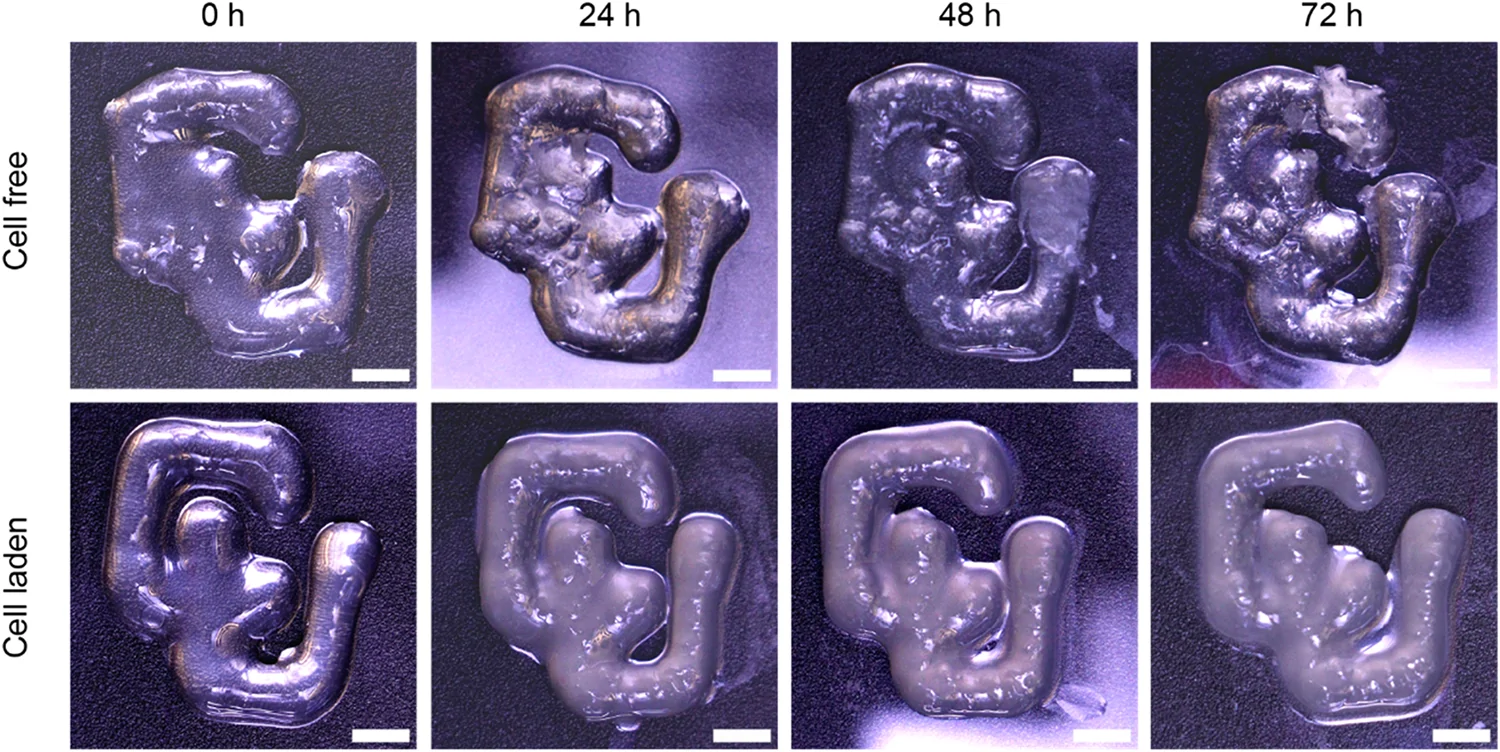
3D printing of the granular hydrogel composites. 3D printing of the CU Boulder logo with cell-free and cell-laden bioink of granular hydrogel composites. Constructs were crosslinked simultaneously during extrusion to enable shape retention. Scale bar are 5 mm.

These outward growth-induced pressures can result in the release of the bacteria. However, this can be an advantage, as ELMs have emerged as a potential delivery platform for probiotics through the encapsulation and release of probiotic bacteria, making sustained bacterial release an important functional characteristic. As such, we demonstrate that our GHCs allow for the controlled release of bacteria. Specifically, we quantified the number of cells released into a surrounding liquid medium over time. We observed an increase in the release of the cells in 24 h and a plateau in cell release after 24 h. We associated this initial increase with rapid colony expansion, fracturing the local hydrogel network and enabling cell escape, while the later steady release suggests sustained transport through these weakened regions (Figure 5). Similar behavior has been previously observed in colony-driven fracture phenomena in hydrogel composites.^47,48^ The sustained release of the bacteria indicates that the GHC systems could be used as a delivery platform for living probiotics.

**Figure 5:**
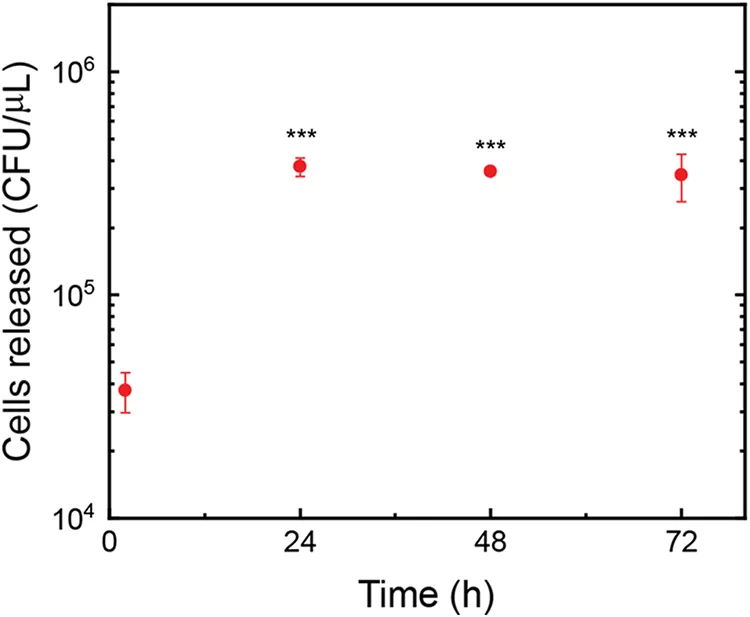
Cell release capabilities of the granular hydrogel composites. Quantification of cell release from granular hydrogel composites as a function of incubation time. All data presented in mean ± standard deviation (*n* = 3). Statistical analysis was performed by a one-way ANOVA with Tukey’s post hoc test and compares each time point to the initial 0 h. *p < 0.05, **p < 0.01, ***p < 0.001.

### 3.5 Patterning of Cellular Chemotaxis

Beyond 3D printing and controlled cell release, GHCs, unlike crosslinked hydrogels, can be leveraged to enable microbial self-organization and patterning before crosslinking. Prior studies have used un-crosslinked microgel systems to investigate the chemotactic migration of bacteria in complex environments.^33,34^ In our platform, the photo-crosslinkable interstitial matrix enables chemotactic self-organization prior to crosslinking and the ability to lock the resulting spatial patterns in place. To demonstrate the stabilization of a spatial pattern, we printed a vertical line of dense cells into the un-crosslinked granular composite (Figure 6A). Initially, the cells maintained a uniform line. After 6 h, a faint halo on the edge of the line emerged, marking the onset of collective migration of microbes. Over the next 12 to 18 h, this wavefront broadened, and by 24 h, a distinct, well-defined chemotactic front formed. Such wave-like expansion indicates collective bacterial chemotaxis, where cells navigate along self-generated nutrient gradients.^33^

**Figure 6:**
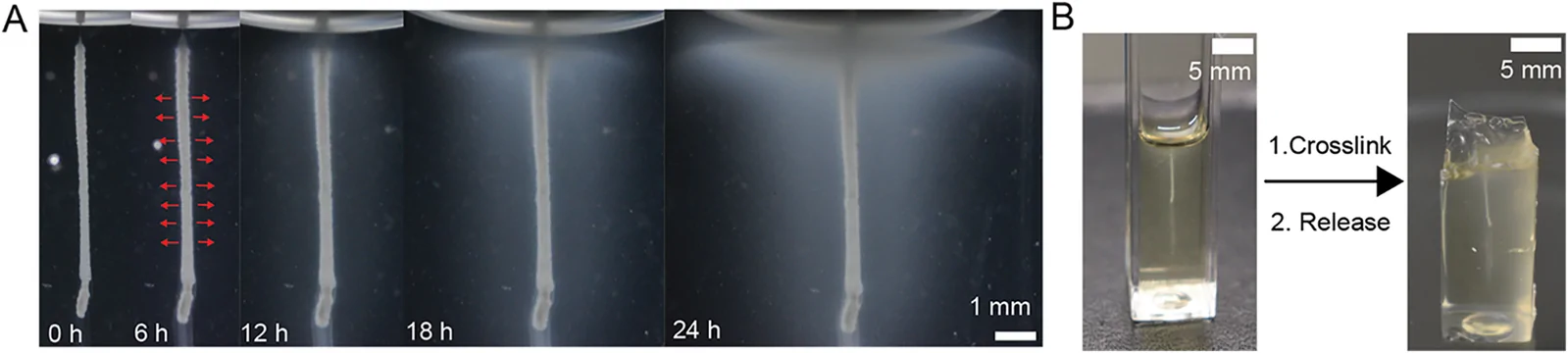
Chemotaxis patterning capability of the granular hydrogel composite. (A) Time-lapse images showing the directional collective cell migration of *E. coli* from an initially printed line, resulting in the progressive formation of a spatial cell density gradient within the granular hydrogel, indicating progressive chemotactic response along a defined path in the granular hydrogel. (B) Representative images of the final structure before and after photo-crosslinking. Before crosslinking, cells had migrated through the un-crosslinked hydrogel while confined within the cold (cuvette). Following photo-crosslinking and removal from the mold, the construct retained its geometry while preserving the biologically generated cell distribution, demonstrating the ability to capture and stabilize emergent microbial patterns.

In this system, local nutrient depletion near the vertical line of dense cells drives outward migration, while the porous microgel network provides sufficient permeability for cell movement (Video S1). After 24 h, we photo-crosslinked the composite, successfully locking the chemotactic wavefront in place (Figure 6B). While not studied in this work, we expect, based on simulations of the chemotactic wavefront in confined media, that there will be a single cell density gradient within the composite.^34^ Thus, we expect that this single cell density gradient could lead to biologically patterned stiffness gradients in the GHC. In the future, GHCs provide a modular, printable platform that integrates microbial growth and collective migration within mechanically stable structures with biologically patterned stiffness gradients to create functionally graded polymers for soft robotics or other ELM applications.

## 4 Conclusions

We developed granular hydrogel composites composed of microgels, a crosslinkable polymer, and *E. coli*, which can be crosslinked when desired with light. We demonstrated that matrix composition impacted bacterial colony morphology, with the granular hydrogel composite promoting larger colonies and more irregular colony morphologies than hydrogels alone. The timing of crosslinking further influenced the spatial distribution of the bacteria and the mechanical response of the composite. To demonstrate potential applications, we showed that the composites can be 3D printed into specified geometries and used for sustained release of bacteria. The composites provide the opportunity to biologically pattern gradients of cells within the composites. Overall, this work establishes how matrix composition, growth environment, bacteria distribution at the time of immobilization can be leveraged to control the compressive modulus of ELMs.

## Supplementary Material





## Notes

Characterization of granular hydrogel composites, including microgel properties, pore size, bacterial colony morphology and distribution, compressive mechanical properties, and 3D-printing fidelity (PDF)
Time-lapse video of directional *E. coli* migration and cell-density gradient formation within the granular hydrogel (MP4)

## Abbreviations

MeHA: methacrylated hyaluronic acid
ELMs: engineered living materials
*E. coli*: *Escherichia coli*
UV: ultraviolet
