# Supplementary material for: Granular Hydrogel Composites as Matrices for Engineered Living Materials
Source: ACS Appl Polym Mater. 2026 Sep 25;8(19):18000–9. doi: 10.1021/acsapm.6c03807 (PMC13660352; doi:10.1021/acsapm.6c03807)
Supplement: Supplementary file 1 [file ap6c03807_si_001.pdf]

Supporting Information

Granular hydrogel composites as matrices for engineered living materials

*Samson Oluwagbenga Adelani [a], Morgan B. Riffe[a], Jason A. Burdick[a,b,c], R. Kōnane Bay [a,b]**

[a]Materials Science and Engineering Program, University of Colorado; Boulder, CO 80303, USA.

[b]Department of Chemical and Biological Engineering, University of Colorado; Boulder, CO 80303, USA.

[c]BioFrontiers Institute, University of Colorado; Boulder, CO 80303, USA.

*Corresponding author: konane.bay@colorado.edu

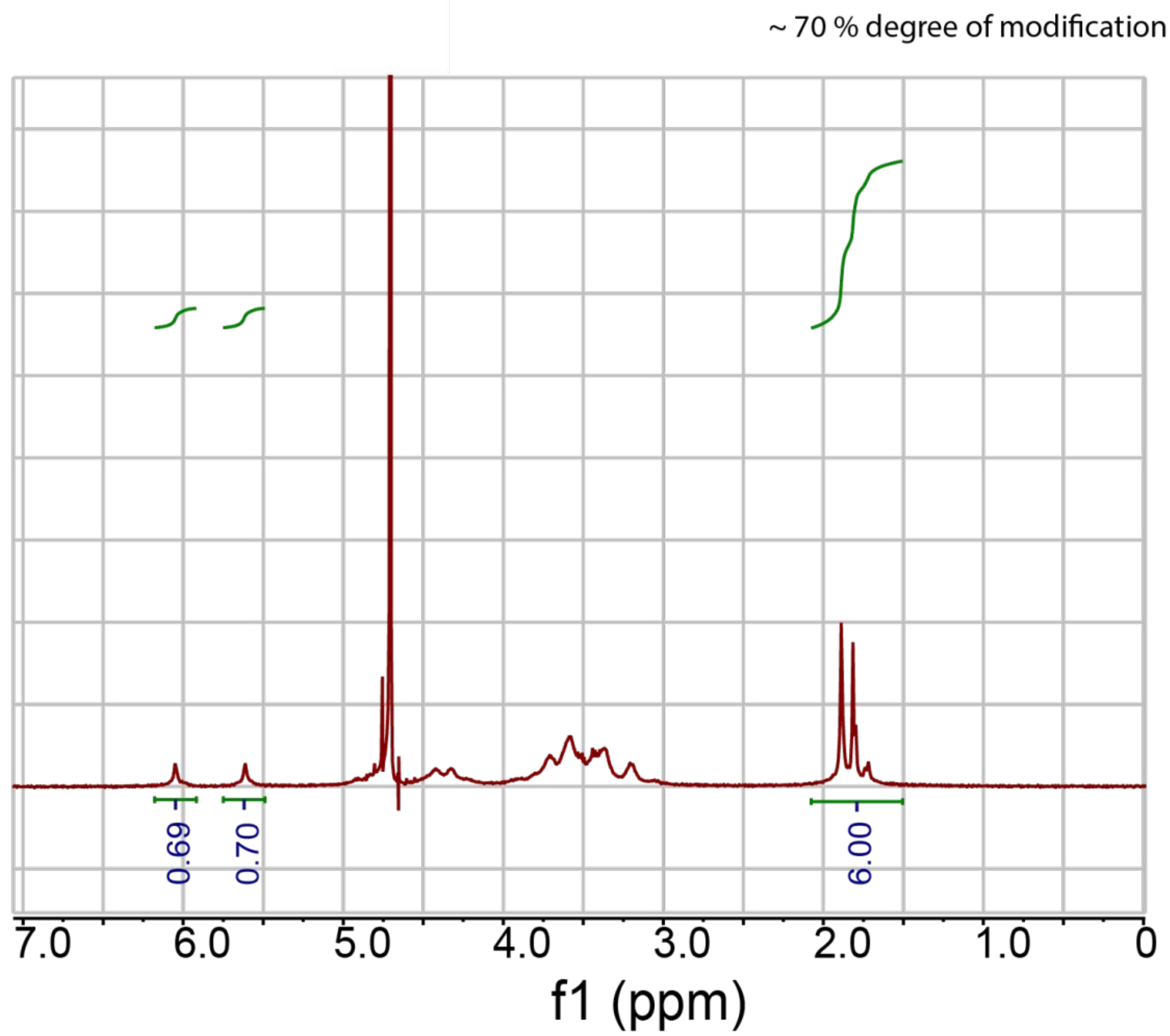

Figure S1. ^{1}H NMR spectrum of methacrylated hyaluronic acid (MeHA) in D_2O showing the methacrylate vinyl proton signals along with the HA backbone peaks. The degree of modification was determined to be ~70%.

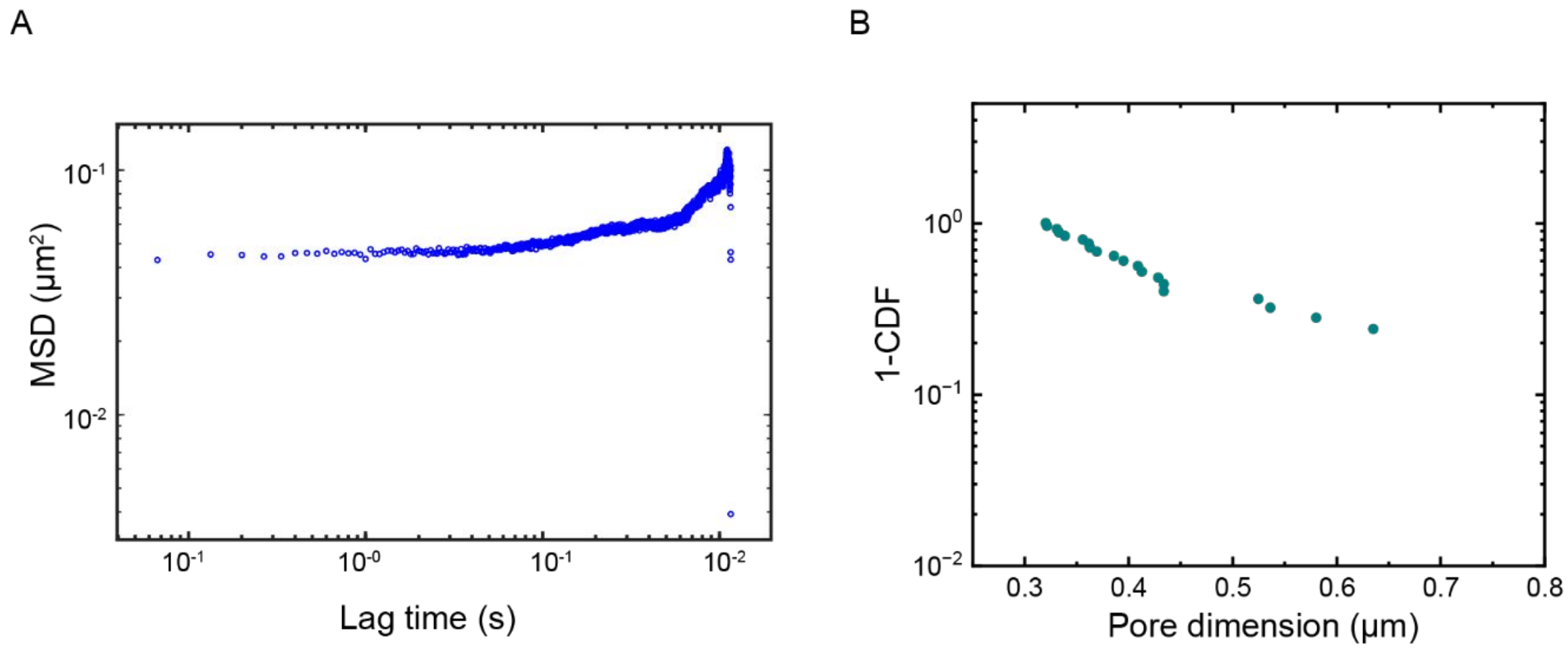

Figure S2. (A) Representative mean-squared displacement (MSD) of a tracer particle. The plateau in the MSD represents confinement of the tracer particle by the surrounding hydrogel microstructure and was used to estimate the characteristic pore size. (B) Pore size distribution analysis of granular hydrogel composites containing 0.7 wt.% microgel (mean pore size: 0.58 ± 0.35 μm, characteristic pore size: 1.8 μm) with 3 wt.% uncrosslinked MeHA. We fit an exponential function B*exp(-a/A) to the 1-CDF data in the range of 0 < CDF < 1. We report the characteristic pore size as ($A+a_0$), where a_0 is the largest pore size with CDF = 0.

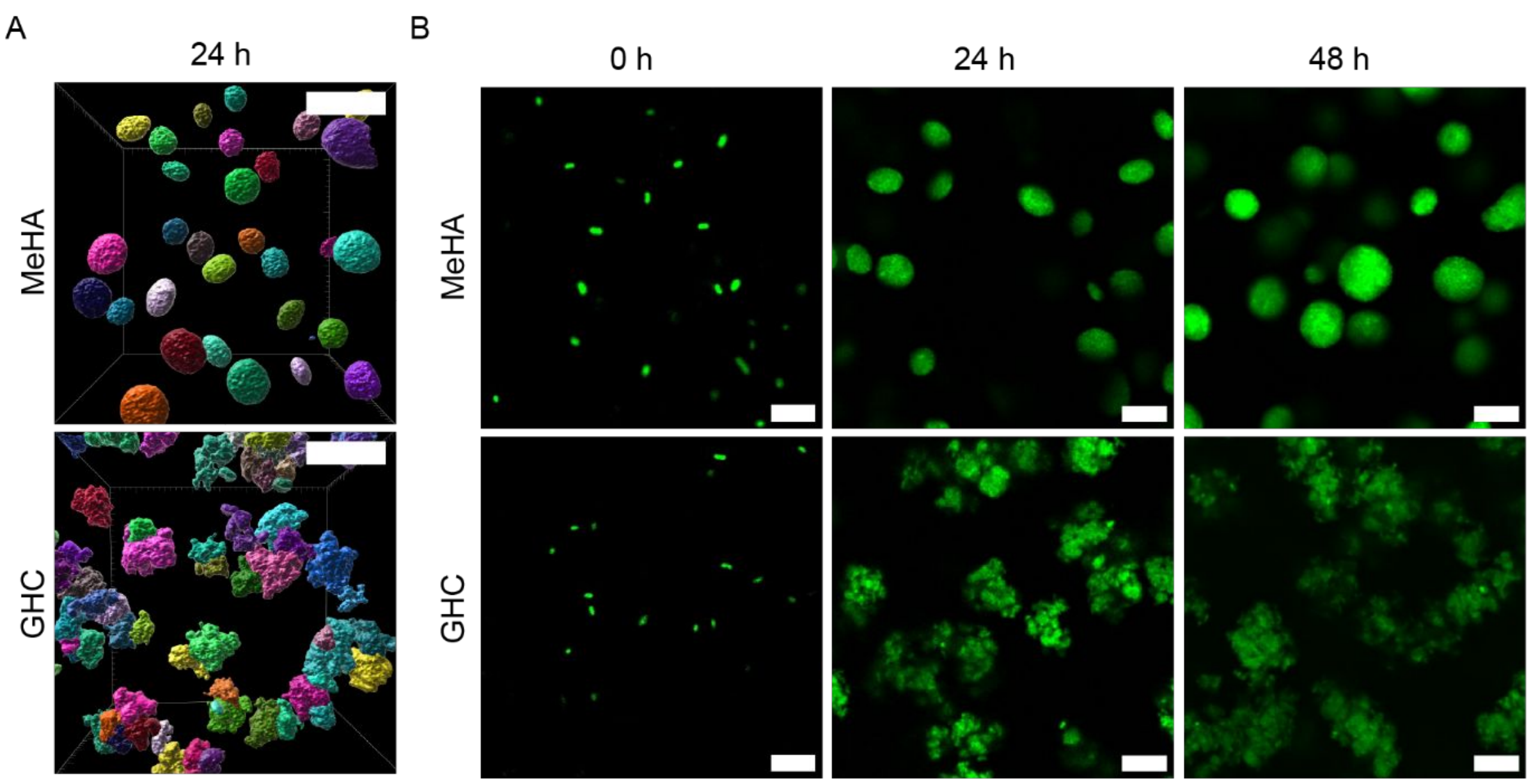

Figure S3. Representative three-dimensional reconstructions of the corresponding bacterial colonies generated from confocal z-stacks using Imaris at 24 h. Individual colonies are displayed as segmented surfaces to visualize colony morphology and growth. Scale bars = 20 μm. (B) Representative confocal fluorescence images of *E. coli* colonies encapsulated within MeHA and GHC from 0 to 48 h of incubation. Colonies cultured in GHCs exhibited significantly larger volumes than those cultured in homogeneous MeHA hydrogels 0 to 48 h. Scale bars = 10 μm.

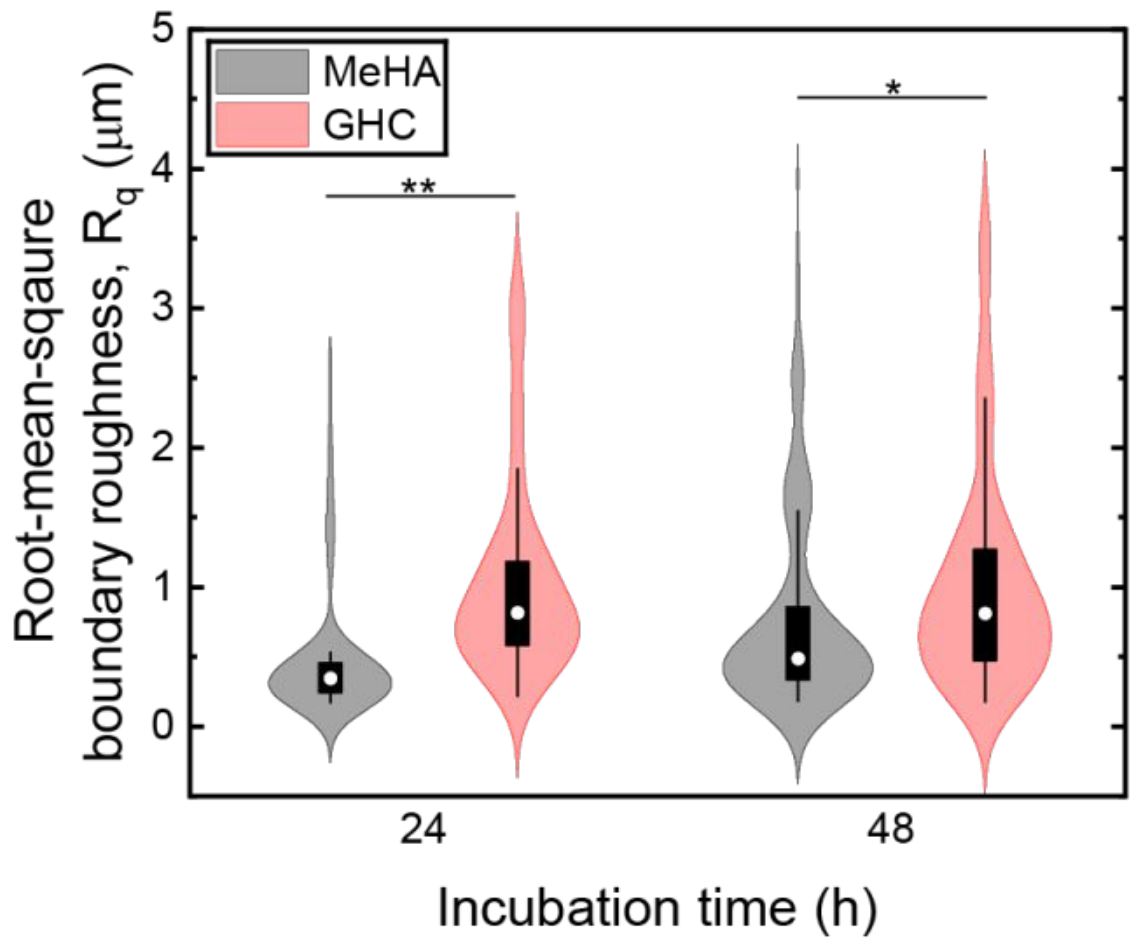

Figure S4. Quantification of Root-mean-square boundary roughness (Rq) of bacterial colonies after 24 and 48 h of incubation. Colonies cultured in GHCs exhibited significantly greater Rq values than colonies cultured in homogeneous MeHA hydrogels at both time points, indicating increased boundary irregularity within the granular matrix. Violin plots represent the distribution of individual colony measurements. Box plots show the interquartile range with the median indicated by the white circle; whiskers represent the 5th and 95th percentiles. Statistical significance was determined by one-way ANOVA with Tukey's multiple. (94 ≥ Number of colonies ≥ 345 from 3 individual experiments, p-values are calculated using one-way ANOVA with post hoc Tukey's test, * $p < 0.05$, ** $p < 0.01$, *** $p < 0.001$).

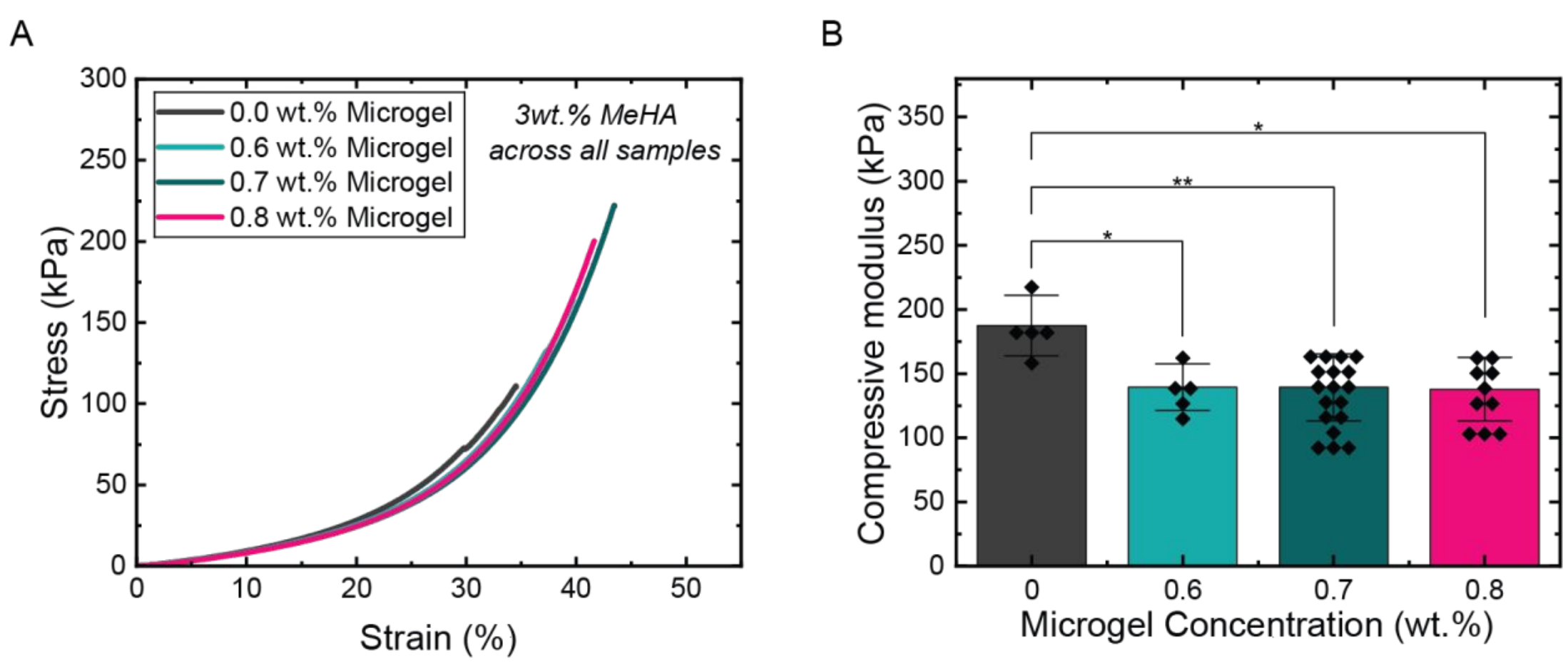

Figure S5. (A) Representative compressive stress–strain curves of cell-free granular hydrogel composites containing 0, 0.6, 0.7, and 0.8 wt.% microgels with a constant MeHA concentration of 3 wt.%. (B) Compressive modulus of cell-free composites as a function of microgel concentration. The addition of microgels reduced the compressive modulus relative to homogeneous MeHA hydrogels. Data are presented as mean ± standard deviation with individual measurements overlaid. Statistical significance was determined using one-way ANOVA followed by Tukey's post hoc test ($p < 0.05$, $p < 0.01$, $p < 0.001$).

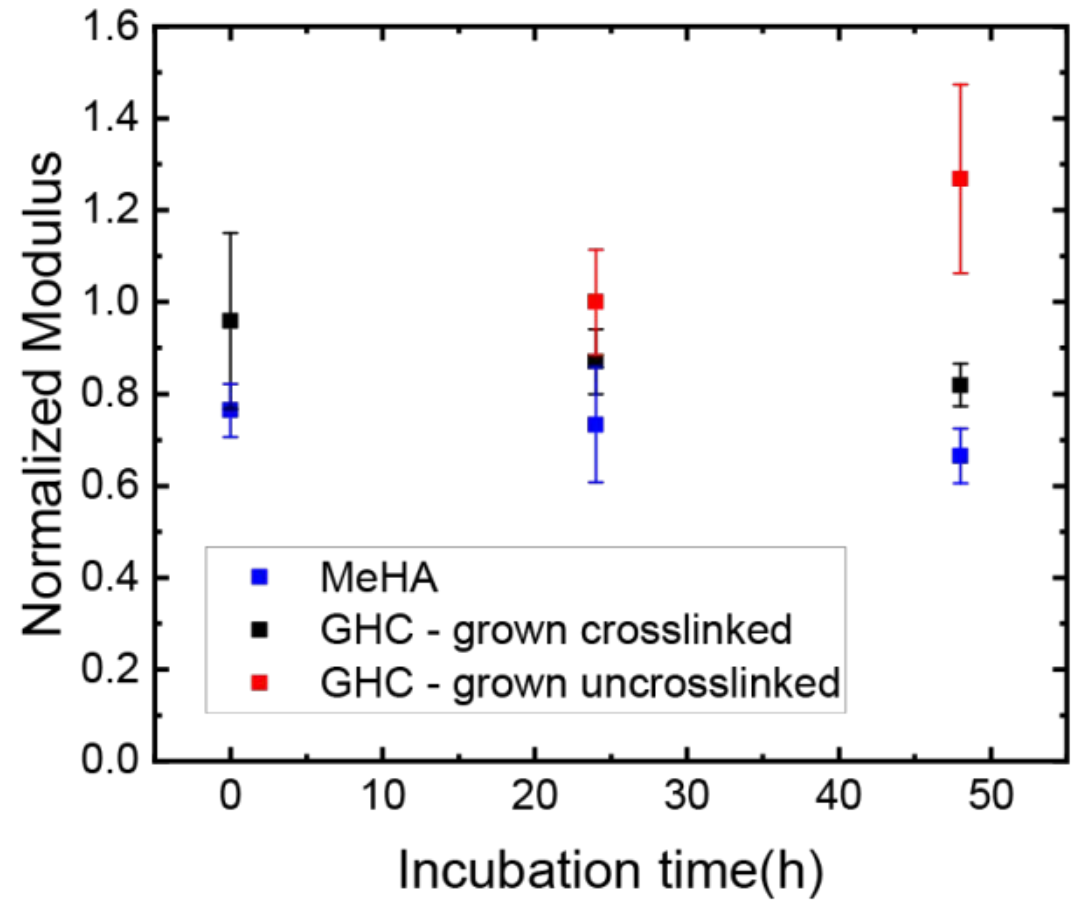

Figure S6. Normalized compressive modulus as a function of incubation time for MeHA, GHC grown crosslinked and GHC grown uncrosslinked prior to photocrosslinking . The compressive modulus was normalized to the corresponding 0 h condition. Compression testing was performed using n = 3–5 independent hydrogel samples per condition, while confocal microscopy analyses were performed using n = 3 independent hydrogel samples per condition. Data are presented as mean ± standard deviation.

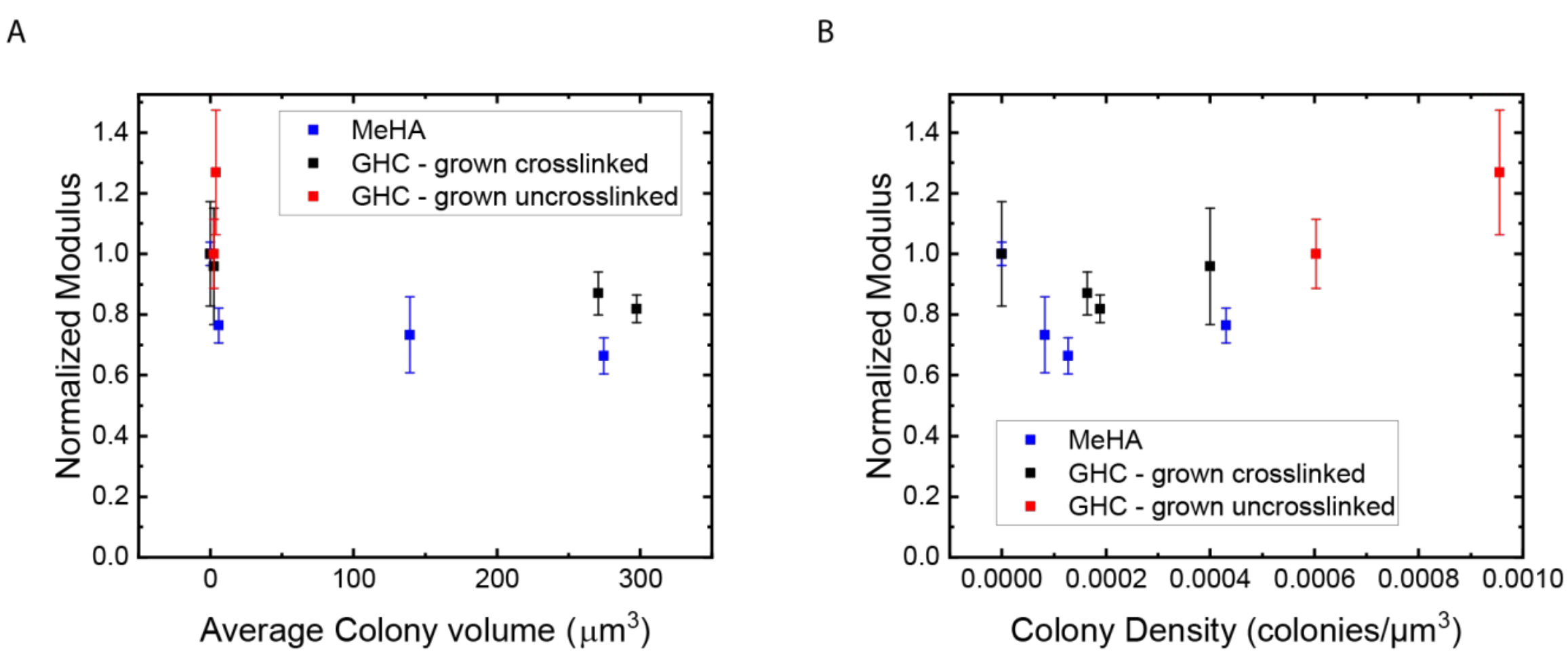

Figure S7. (A) Normalized compressive modulus as a function of colony volume for MeHA, GHC grown crosslinked and GHC grown uncrosslinked prior to photocrosslinking (B) Normalized compressive modulus as a function of colony density (number of colonies per unit sample volume. Data are presented as mean ± SD. Compression testing was performed using n = 3–5 independent hydrogel samples per condition, while confocal microscopy analyses were performed using n = 3 independent hydrogel samples per condition. Data are presented as mean ± standard deviation.

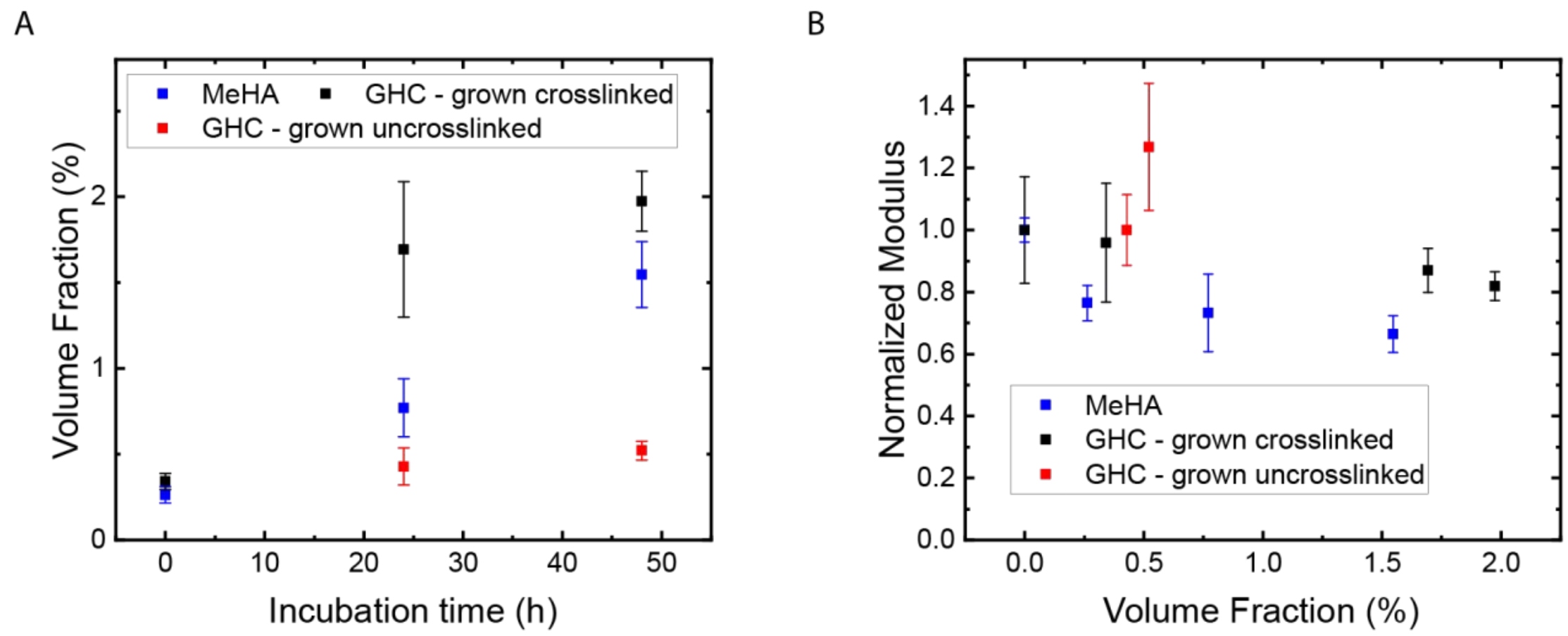

Figure S8. (A) Volume fraction as a function of incubation time for MeHA, GHC grown crosslinked and GHC grown uncrosslinked prior to photocrosslinking (B) Normalized compressive modulus as a function of bacterial volume fraction. Compression testing was performed using n = 3–5 independent hydrogel samples per condition, while confocal microscopy analyses were performed using n = 3 independent hydrogel samples per condition. Data are presented as mean ± standard deviation.

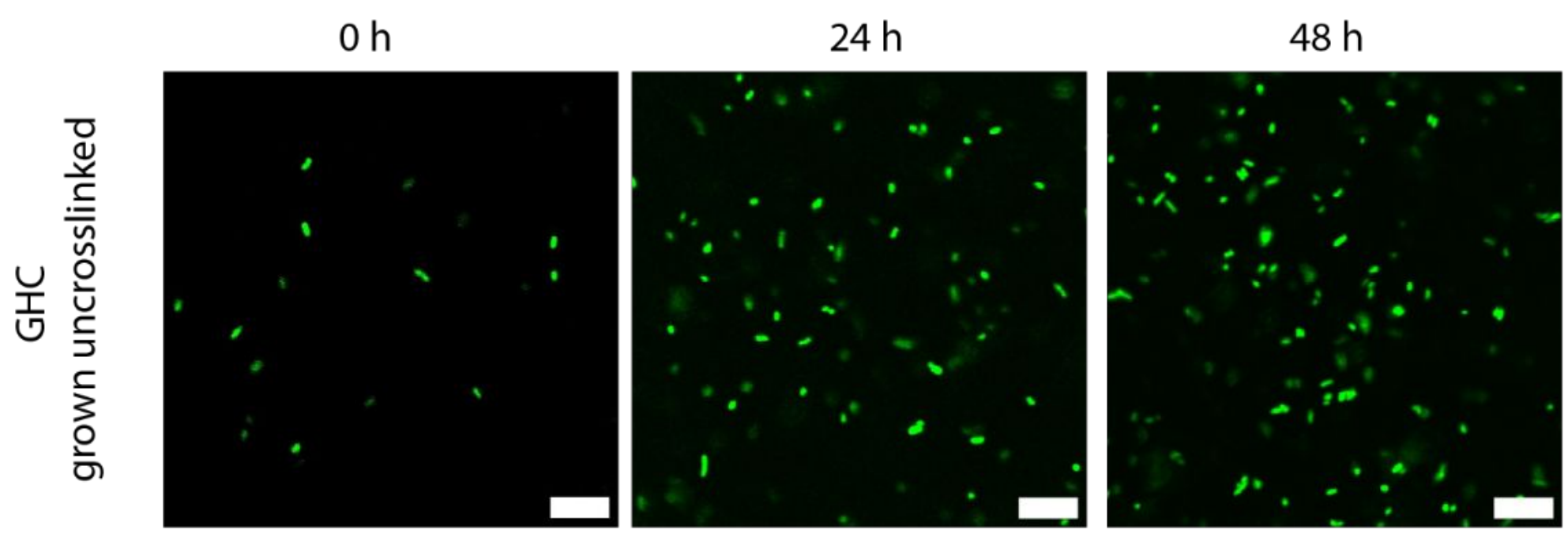

Figure S9. Representative confocal fluorescence microscopy of *E. coli* colonies encapsulated within GHC grown uncrosslinked from 0 to 48 h. Scale bars are 10 µm.

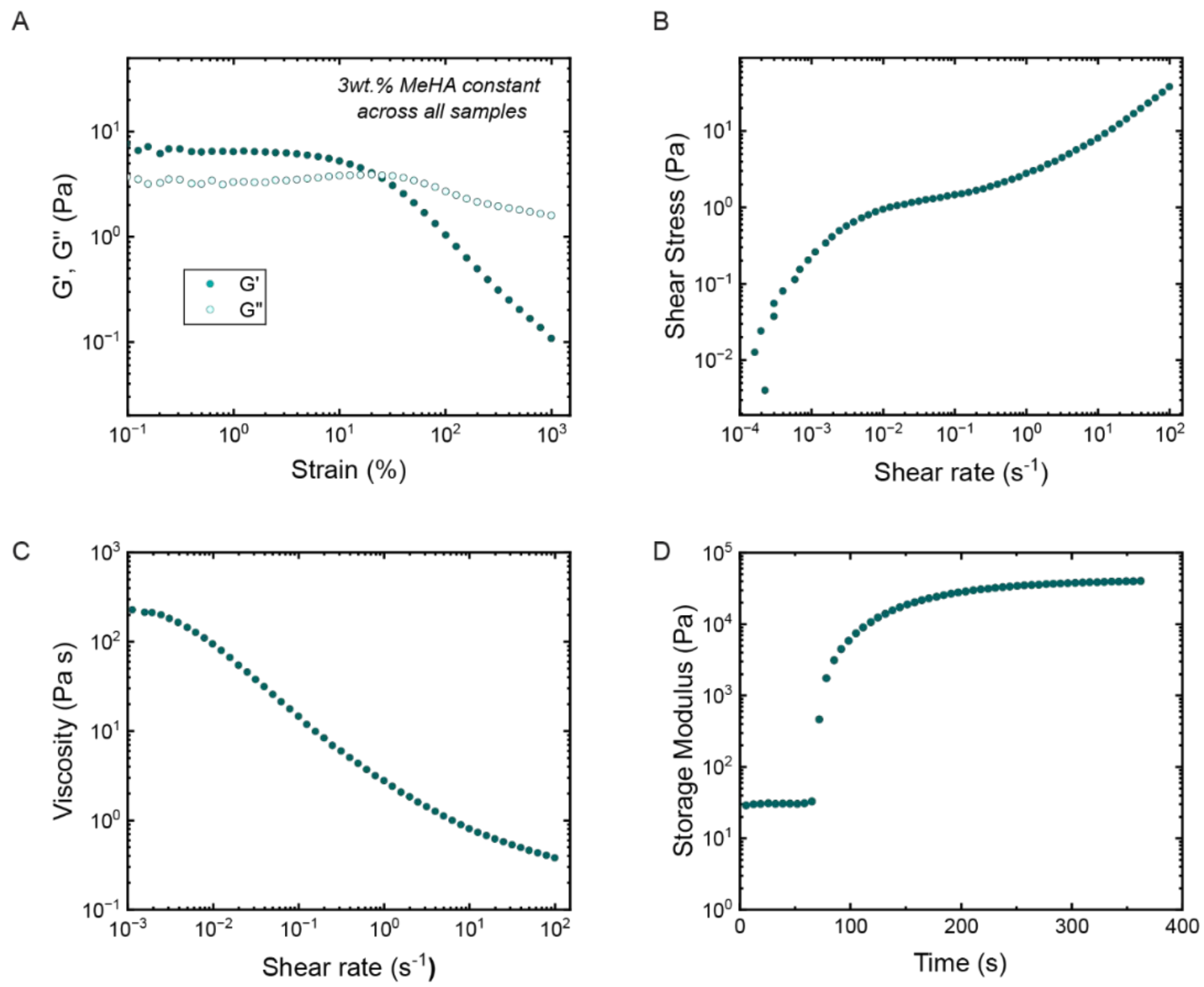

Figure S10. Rheological characterization of granular hydrogel composites with 0.7 microgel (3 wt.% MeHA), including (A) strain sweep, (B) flow curve (shear stress vs. shear rate), (C) viscosity vs. shear rate, and (D) photorheology showing storage modulus during UV crosslinking.

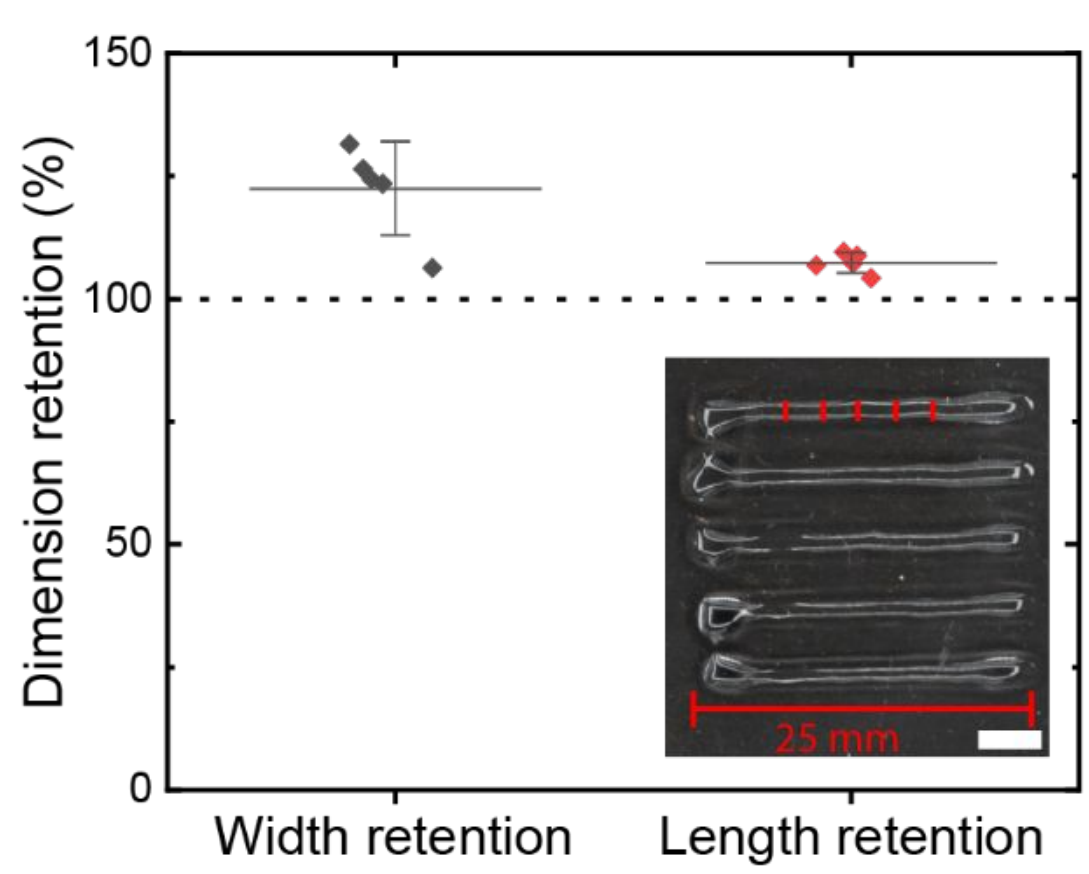

Figure S11. Representative printed lines are shown with a 5 mm scale bar. Filament width and length were normalized to the 20G needle inner diameter (0.660 mm) and programmed length (25 mm), respectively. Individual points represent printed filaments (n = 5); data are mean ± SD. The dashed line indicates 100%-dimensional retention.
